# Abnormal expression of *TRIB3* in colorectal cancer: a novel marker for prognosis

**DOI:** 10.1038/sj.bjc.6605361

**Published:** 2009-11-10

**Authors:** N Miyoshi, H Ishii, K Mimori, Y Takatsuno, H Kim, H Hirose, M Sekimoto, Y Doki, M Mori

**Affiliations:** 1 Department of Gastroenterological Surgery, Osaka University Graduate School of Medicine, Suita, Yamadaoka 2-2, Osaka 565-0871, Japan; 2 Division of Molecular and Surgical Oncology, Department of Molecular and Cellular Biology, Kyushu University, Medical Institute of Bioregulation, Tsurumihara 4546, Beppu, Ohita 874-0838, Japan

**Keywords:** *TRIB3*, prognosis, metastasis, colorectal cancer

## Abstract

**Background:**

*TRIB3* is a human homologue of *Drosophila tribbles*. Previous studies have shown that *TRIB3* controls the cell growth through ubiquitination-dependent degradation of other proteins, whereas its significance in the prognosis of colorectal cancer (CRC) is not yet fully understood.

**Materials:**

This study comprised 202 patients who underwent surgery for CRC, as well as 22 cell lines derived from human gastrointestinal cancer. The correlation of gene expression with clinical parameters in patients was assessed. The biological significance was evaluated by knockdown experiments in seven colorectal cancer cell lines.

**Results:**

A total of 20 cancer cell lines (90.9%) expressed the *TRIB3* gene. The assessment in surgical specimens indicated that the gene expression was significantly higher in the cancerous region than in the marginal non-cancerous region. Patients with high *TRIB3* expression were statistically susceptible to a recurrence of the disease, and showed poorer overall survival than those with low expression. The assessment of *TRIB3* knockdown in five cell lines showed that small interfering RNA (siRNA) inhibition resulted in a statistically significant reduction in cell growth.

**Conclusion:**

These data strongly suggest the usefulness of *TRIB3* as a marker for predicting the prognosis of CRC patients, showing a basis for the development of effective treatments for CRC.

In many developed countries, including the United States and Japan, cancer is one of the most prominent illnesses in public welfare and health measures (Jones *et al*, 2007; Jemal *et al*, 2008). The incidence of colorectal cancer (CRC) has increased significantly in recent years, in concert with the changing lifestyle (Kohno *et al*, 2007). The major cause of death in CRC is liver metastases (Yamasaki *et al*, 2007). Although treatment of CRC has improved recently, it fails in approximately one-third of the patients who need an alternative strategy for coping with death (Jones *et al*, 2007). In this matter, useful predictive markers would be desired in the medication of CRC patients.

As shown in other tumours, tumour-promoting oncogenes and tumour suppressors control cell proliferation through cell-cycle arrest of CRC (Aliaga *et al*, 1999; Jemal *et al*, 2008; Yamatodani *et al*, 2009). Further identification of genes responsible for the development and progression of CRC, as well as understanding of their clinical significance, would lead to efficient diagnosis and treatment of the disease. Characterization of key molecules is particularly promising for the development of new approaches for the treatment of gastrointestinal tumours.

Previous studies have shown that chromosomal aberrations occur during carcinogenesis, and relate to patients’ prognoses in CRC (Hermsen *et al*, 2002; Leslie *et al*, 2003). Alterations of particular loci at chromosome 20 are reported, indicating the significance of studies on this chromosomal region (Wang *et al*, 2001; Pledgie *et al*, 2005; Yde *et al*, 2007; Goodwin *et al*, 2008; Shor *et al*, 2008). It has been shown that aberrant gains at chromosome 20 are specifically associated with mutations in the tumour suppressor gene, *TP53*, by a survey of 50 cases of CRC, and they are also correlated with the progression of CRC, suggesting that the tumour suppressor pathway is involved in the maintenance of particular chromosomal regions (Wang *et al*, 2001; Leslie *et al*, 2003; Pledgie *et al*, 2005; Yde *et al*, 2007; Goodwin *et al*, 2008; Shor *et al*, 2008).

Although previous studies suggest candidate genes in the regions at chromosome 20, which might have a role in CRC, it is yet to be fully understood in prognostic value (Wu *et al*, 2006; Zheng *et al*, 2008; Antonacopoulou *et al*, 2008). Here we report on *TRIB3* gene in the chromosomal region at 20p13, which is overexpressed in CRC, as a new marker for prognosis and metachronous metastasis. Trib3 is a human homologue of *Drosophila tribbles 3*, which regulates cell growth, differentiation, oogenesis and metabolism by promoting ubiquitination-dependent degradation of other proteins, interacts with several transcriptional factors and is expressed in several tumours (Mata *et al*, 2000; Bowers *et al*, 2003; Du *et al*, 2003; Koo *et al*, 2004; Boudeau *et al*, 2006; He *et al*, 2006; Koh *et al*, 2006; Matsushima *et al*, 2006; Ord *et al*, 2007; Kato and Du, 2007; Xu *et al*, 2007; Yao and Nyomba, 2008). We studied the *TRIB3* gene in 202 paired cancerous and non-cancerous regions of CRC, as well as 7 colorectal cancer cell lines and 15 other gastrointestinal cancer cell lines. Our data indicate the clinical significance of *TRIB3* in the evaluation of CRC prognosis.

## Materials and methods

### Cell lines and culture

A total of 22 cell lines derived from human CRC and other gastrointestinal cancer (for CRC: Caco2, DLD-1, LoVo, HCT116, HT-29, KM12SM and SW480; for oesophageal cancer: TE-5, TE-8 and TE-10; for gastric cancer: MKN28 and MKN45; for pancreatic cancer: MIAPaCa-2, PANC-1 and PSN-1; for hepatocellular carcinoma: HuH-7, HepG2, Hep3B, HLE, HLF and PLC; for cholangiocellular carcinoma: HuCCT-1) were maintained in Dulbecco's modied Eagle's medium containing 10% fetal bovine serum and antibiotics at 37°C in a 5% humidified CO_2_ atmosphere. For small interfering RNA (siRNA) inhibition, double-stranded RNA duplexes targeting human *TRIB3* (5′-GCGGUUGGAGUUGGAUGACAACUUA-3′ and 5′-GCGUGAUCUCAAGCUGUGUCGCUUU-3′) were purchased as a Validated Stealth RNAi kit (Invitrogen, Carlsbad, CA, USA), as well as negative control siRNA (12935-112, Stealth RNAi Negative Control, Medium GC Duplex, Invitrogen). CRC cell lines were transfected with siRNA at a concentration of 20 *μ*mol ml^−1^ using lipofectamine RNAiMAX (Invitrogen), incubated in glucose-free Opti-MEM (Invitrogen) and analysed using CellTac, a proliferation assay kit (Invitrogen). Values are presented as means±s.d. from all independent experiments performed in triplicate.

### Clinical tissue samples

The study comprised 202 patients who underwent surgery for CRC, including 118 patients at Kyusyu University from 1992 to 2002, and 84 patients at Osaka University from 2002 to 2006. Primary CRC specimens and adjacent normal colorectal mucosa were obtained from patients after written informed consent had been confirmed, in accordance with institutional ethics guidelines. The surgical specimens were fixed in formalin, processed through graded ethanol and embedded in paraffin, and were sectioned with haematoxylin and eosin staining (see the Supplementary Information). For RNA study, all specimens were frozen immediately after resection in liquid nitrogen and were kept at −80°C until RNA extractions. None of the patients received chemotherapy or radiotherapy before surgery. After surgery, patients were followed up with blood examinations including those for tumour markers, such as serum carcinoembryonic antigen and cancer antigen (CA19-9), and imaging modalities such as abdominal ultrasonography, computed tomography and chest X-ray every 3–6 months. Clinico-pathological factors were assessed according to the criteria of tumour–node–metastasis (TNM) classification of the International Union Against Cancer (UICC) (Sobin and Fleming, 1997).

### RNA preparation and reverse transcriptase PCR (RT–PCR)

Total RNA was prepared using TRIzol reagent (Invitrogen) or with DNase by a modified acid guanidium–phenol–choroform procedure (Mimori *et al*, 1997). Reverse transcription was performed with SuperScriptII (Invitrogen) or with 2.5 *μ*g of total RNA as previously described (Mori *et al*, 1993). A 158-bp *TRIB3* fragment was amplified. Two human *TRIB3* oligonucleotide primers for the PCR reaction were designed as follows: 5′-TGCCCTACAGGCACTGAGTA-3′ (forward); 5′-GTCCGAGTGAAAAAGGCGTA-3′ (reverse). The forward primer is located in exon 2 and the reverse primer in exon 3. To confirm PCR amplification, 25–35 cycles of PCR reaction were performed using a PCR kit (Takara, Kyoto, Japan) on a Geneamp PCR system 9600 (PE Applied Biosystems, Foster City, CA, USA) with the following parameters: 95°C for 10 s, 60°C for 10 s and 72°C for 60 s. An 8-*μ*l aliquot of each reaction mixture was size-fractionated in a 1.5% agarose gel and visualised using ethidium bromide staining. To confirm RNA quality, a PCR amplification of 270 bp was performed for the glyceraldehyde-3-phosphate dehydrogenase (*GAPDH*) gene using the following primers: 5′-TTGGTATCGTGGAAGGACTCA-3′ and 5′-TGTCATCATATTGGCAGGTT-3′. Human reference complementary DNAs were used as positive controls (Clontech).

### Quantitative real-time RT–PCR

For quantitative assessment, quantitative real-time RT–PCR was performed using a kit, LightCycler FastStart DNA Master SYBR Green I (Roche Diagnostics, Tokyo, Japan), for PCR amplification of *TRIB3* and *GAPDH*. The amplification protocol consisted of denaturation at 95°C for 10 s, annealing at 60°C for 10 s and elongation at 72°C for 10 s. The products were then subjected to a temperature gradient from 55 to 95°C with continuous fluorescence monitoring to produce a melting curve of the products. The expression ratios of mRNA copies in tumour and normal tissues were calculated to normalise against *GAPDH* mRNA expression.

### Immunohistochemistry

A total of 20 cases of CRC surgical specimens from formalin-fixed, paraffin-embedded tissues were used for Trib3 immunohistochemistry. After deparaffinization and blocking, the antigen–antibody complex was incubated overnight at 4°C. ENVISION reagents (Dako Cytomation, Glostrup, Denmark) were used to detect the signal from the antigen–antibody reaction. All sections were counterstained with haematoxylin. The primary anti-Trib3 rabbit polyclonal antibody (HPA015272; Sigma, St Louis, MO, USA) was used at a dilution of 1 : 100. All sections were independently examined for protein expression, and assessed by comparison of staining between normal and cancer regions under microscopic examination of ⩾100 fields in each specimen.

### Proliferation assay

To determine the proliferative properties, 1.0 × 10^5^ cells were seeded and cultured into each 24-well dish. The cell growth rate was measured by counting cells using a CellTac kit (Nihon Koden, Tokyo, Japan).

### Statistical analysis

For continuous variables, data are expressed as mean±s.d. The relationship between *TRIB3* expression and clinico-pathological factors was analysed using χ^2^ and Student's *t*-tests. Kaplan–Meier survival curves were plotted and compared with the generalised log-rank test. Univariate and multivariate analyses for the identification of prognostic factors were performed using a Cox proportional hazard regression model. All tests were analysed using JMP software (SAS Institute, Cary, NC, USA). Differences with *P*-values <0.05 were considered statistically significant.

## Results

### Expression of TRIB3 in CRC cell lines and clinical tissue specimens

**Figure 1:**
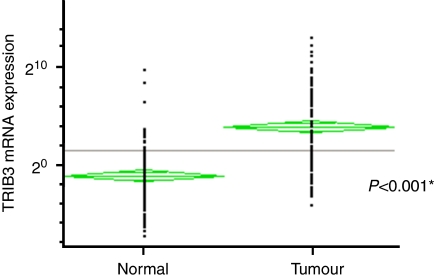
*TRIB3* mRNA expression in clinical tissue specimens. Quantitative real-time RT–PCR on 202 paired clinical samples showed that 181 of 202 (89.6%) samples had higher levels of *TRIB3* mRNA in tumours than in paired normal regions. The mean expression value of *TRIB3* mRNA in tumour regions, 154.62±1021.63 (mean±s.d.; normalised by *GAPDH* gene expression), was significantly higher than the value, 6.98±4.91, for the corresponding normal regions (*P*<0.001; Student's *t*-test). GAPDH=glyceraldehydes-3-phosphate dehydrogenase; RT–PCR= reverse transcriptase PCR; TRIB3=tribbles homologue 3.

We first studied the expression of *TRIB3* gene, and evaluated it in gastrointestinal cancer cell lines and clinical tissue samples by RT–PCR analysis to confirm that the PCR amplification was specific and produced a single band in agarose gel, stained with ethidium bromide, before performing real-time PCR. The RT–PCR study of *TRIB3* in 22 human gastrointestinal cancer lines indicated 20 cells (90.9%; TE-8, TE-10, MKN45, MIAPaCa-2, PANC-1, PSN-1, HuH-7, HepG2, Hep3B, HLE, HLF, PLC, HuCCT-1, Caco2, DLD-1, LoVo, HCT116, HT-29, KM12SM and SW480) that expressed the *TRIB3* gene with a band in gel (the Supplementary Figure S1A). The RT–PCR analysis of *TRIB3* in primary CRC samples was then performed in paired normal and tumour samples (representative data shown in Supplementary Figure S1B: *TRIB3* expression was higher in cancerous regions than in normal regions). Quantitative real-time RT–PCR on 202 paired cancer and normal samples showed that 181 of 202 (89.6%) samples had higher levels of *TRIB3* mRNA in cancerous regions than in normal regions (Figure 1). The mean expression value of *TRIB3* mRNA in cancerous regions (normalised by *GAPDH* gene expression) was significantly higher than the value in the corresponding normal regions (*P*<0.001; Student's *t*-test).

### Expression of Trib3 protein

**Figure 2:**
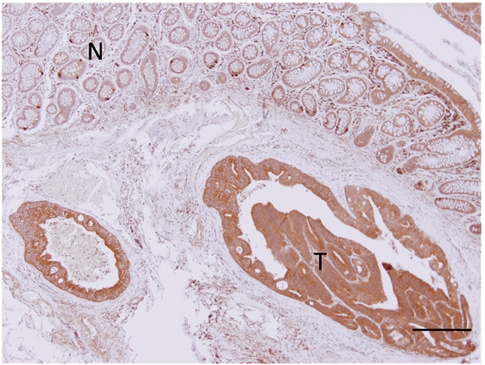
Immunohistochemical staining for Trib3 in tumour and normal specimens. A representative positive stain for Trib3 in tissues from CRC patients. Positive staining is observed in the nucleus and cytoplasm of cancer cells, but not in stromal cells. Trib3 expression was associated with mRNA expression. CRC=colorectal cancer; T=tumour cells; N=normal glandular cells. Bar=200 *μ*m (original magnification, × 20).

Figure 2 shows a representative immunohistochemical staining pattern for Trib3 in tissue from a CRC patient. Trib3 protein staining was observed in the nucleus and cytoplasm in epithelial cells; the expression of CRC was compared with non-cancerous epithelial cells, whereas the expression was appreciably weak or hardly detectable in stromal cells. Examination of 20 cases, which were selected randomly, indicated that 16 cases showed a higher expression level of Trib3 protein in cancerous regions compared with normal regions, whereas the remaining four cases showed no difference between normal and cancerous regions. To compare the data, mRNA expression was assessed by gel RT–PCR and real-time RT–PCR. The data show that mRNA expression was high level in all 16 immunohistochemistry-positive tumours, whereas mRNA expression was comparable in normal and cancerous regions of the remaining four tumours, suggesting that the high expression of Trib3 protein is associated with mRNA expression (*P*<0.001; χ^2^ test). No variation of staining intensity for Trib3 was observed in each of the specimens. We concluded that both mRNA and the protein coded by this gene are associated and frequently expressed together in CRC.

### TRIB3 expression and clinico-pathological characteristics

To study the *TRIB3* expression in CRC quantitatively, the data were classified into two experimental groups on the basis of the *TRIB3* expression levels to assess the expression value without any bias. The high-expression group comprised patients who had a level of *TRIB3* expression higher than the median value for *TRIB3*/*GAPDH* expression in tumour regions compared with normal regions (*n*=101); other patients were assigned to the low-expression group (*n*=101). Clinico-pathological factors related to *TRIB3* expression status are shown in Table 1. Data indicated that metastasis (M0 / M1) was correlated with *TRIB3* expression (*P*<0.001). The metastatic sites were the liver (37 cases), lung (10 cases), brain (3 cases) and bone (1 case). Metastatic sites and other factors were not significantly correlated with *TRIB3* expression.

### Relationship between TRIB3 expression and prognosis

**Figure 3:**
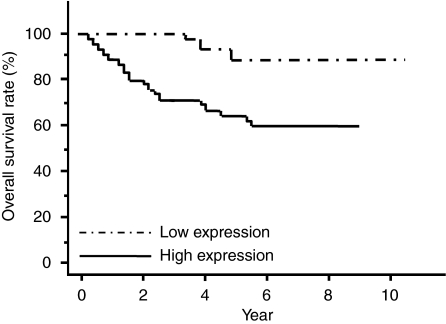
Overall survival rates of patients with CRC on the basis of TRIB3 mRNA expression status. The overall survival rate was significantly lower in the *TRIB3* high-expression group than that in the low-expression group (*P*<0.001). CRC=colorectal cancer; TRIB3=tribbles homologue 3.

The study of prognosis revealed that the overall survival rate was significantly lower for patients in the high-expression group (*P*<0.001; Figure 3). The median follow-up was 2.98 years. Table 2 shows the univariate and multivariate analyses of factors related to patient prognosis. Univariate analysis showed that the post-operative overall survival was significantly correlated with following factors: tumour size (*P*=0.001), tumour invasion (*P*<0.001), lymph node metastasis (*P*=0.001), lymphatic invasion (*P*=0.021), metastasis (*P*<0.001) and *TRIB3* expression (*P*<0.001). Multivariate regression analysis indicated that an inclusion in the *TRIB*3 high-expression group (relative risk (RR)=3.78; 95% confidence interval (CI)=1.27–16.35; *P*=0.014) was an independent predictor of overall survival, as was metastasis (M1 / M0) (RR=9.34; 95% CI=3.70–27.28; *P*<0.001), indicating a significant link between *TRIB3* expression and patient prognosis.

**Figure 4:**
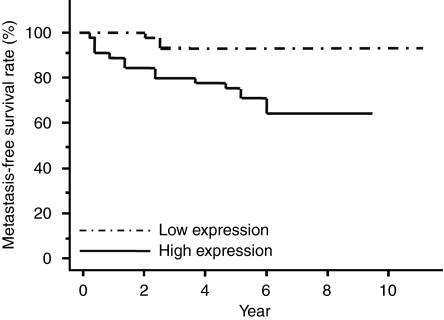
Metachronous metastasis-free over 5 years’ survival rates of patients with CRC in stages I II, and III, on the basis of TRIB3 mRNA expression status. The metachronous metastasis-free over 5 years’ survival rate was significantly lower in patients with the *TRIB3* high-expression group compared with the low-expression group (*P*=0.007). CRC=colorectal cancer; TRIB3=tribbles homologue 3.

In 65 of 202 patients, we have followed up over 5 years after the primary operation, the median follow-up was 6.31 years. We then evaluated the metachronous, metastasis-free, over 5years’ survival in these patients, indicating that the rate was significantly lower in patients of the high-expression group (*P*=0.007, Figure 4). Table 3 shows the univariate and multivariate analyses of factors related to patient prognosis. Univariate analysis showed that the post-operative metastasis was significantly correlated with following factors: histological grade (*P*<0.001), tumour size (*P*<0.001), tumour invasion (*P*=0.003), lymph node metastasis (*P*=0.001), lymphatic invasion (*P*=0.001), venous invasion (*P*=0.019) and *TRIB3* mRNA expression (*P*=0.006). Multivariate regression analysis indicated that inclusion in the *TRIB3* high-expression group (RR=3.86; 95% CI=1.09–19.00; *P*=0.035) was an independent predictor of metastasis-free survival, as were histological grade (RR=25.9; 95% CI=3.57–215.84; *P*=0.001) and tumour size (RR=3.04; 95% CI=1.18–13.62; *P*=0.017).

### Effect of TRIB3 inhibition in CRC cell growth

**Figure 5:**
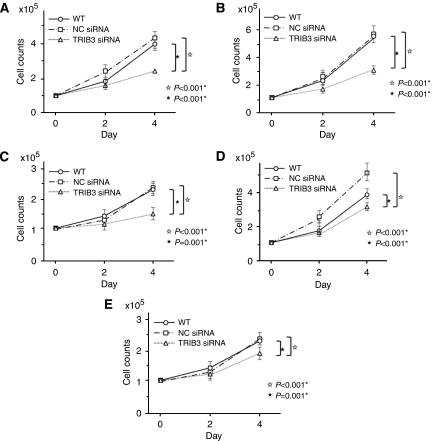
Proliferation assay with siRNA inhibition in five CRC cell lines. Proliferation assay was performed in five CRC cell lines (**A**, DLD-1; **B**, LoVo; **C**, HCT-116; **D**, KM12SM; E, SW480). There were significant differences between WT or NC, and *TRIB3* siRNA. Values are presented means±s.d. of three independent experiments. CRC=colorectal cancer; NC=negative control; TRIB3=tribbles homologue 3; WT=wild type.

A total of 7 CRC cell lines were subjected to siRNA knockdown. The biological role of *TRIB3 in vitro* was analysed in CRC, in which *TRIB3* expression was knocked down. In the CRC cell lines examined, significant suppression of endogenous *TRIB3* expression by siRNA was confirmed by real-time RT–PCR in five cell lines (DLD-1, LoVo, HCT116, KM12SM and SW480; *P*<0.05, Student’s *t*-test; Supplementary Figure S2). To determine the proliferative properties, cells were seeded and cultured (Figure 5). There were significant differences in numbers between wild-type or negative control and *TRIB3* siRNA (*P*<0.05) in all five CRC cell lines. There was no significant change in number between negative control and wild type.

## Discussion

This study showed that *TRIB3* is expressed at higher levels in CRC than in the corresponding normal regions, and is expressed in gastrointestinal cancer cell lines. The siRNA inhibition experiment showed the functional relevance of expressed *TRIB3* in gastrointestinal cancer cell lines. To the best of our knowledge, this study is the first to show the candidacy of *TRIB3* as a prognostic CRC marker, supported by the functional relevance to cell growth.

Nowadays, it can be useful to determine the necessity of intensive follow-up and adjuvant therapy for CRC by predicting recurrence and metastases in curative surgical resection (Bathe *et al*, 2004; Kornmann *et al*, 2008; Wolpin and Mayer, 2008). In this study, clinico-pathological analysis revealed that TRIB3 is closely related to metastasis, but not to lymphatic metastasis. It may correlate with some mechanism of little concern to invasiveness. Patients with CRCs with high *TRIB3* expression showed a poorer prognosis for disease-free and overall survival than those in the low-expression group. Data indicate that *TRIB3* is an independent prognostic factor, as well as a very important predictor that is already known (Derkinderen *et al*, 1990). *TRIB3* is presumably a good predictor of metachronous metastasis that can be followed by curative surgical intervention. In gastrointestinal cancer therapy, it is essential to prevent metachronous metastasis. Several adjuvant chemotherapies are helpful in certain disease stages, especially in CRC (Bathe *et al*, 2004; Andre *et al*, 2007). Recently, increasing evidence has been accumulated, showing the usefulness of less invasive surgery in the treatment of CRC, such as laparoscopic and endoscopic surgery (Lacy *et al*, 2002; Weeks *et al*, 2002; Clinical Outcomes of Surgical Therapy Study Group, 2004; Jayne *et al*, 2007). For these cases, predictive markers of tumour invasion and metastasis, which are independent of traditional TNM classification and contribute collectively to diagnoses and treatments, are very important. These data indicate the candidacy of *TRIB3*.

Although improving treatments such as pre-operative and post-operative chemotherapy and radiotherapy combined with surgery for CRC have contributed to the reduction of recurrences and metastases, half of the cases eventually metastasise despite systemic chemotherapy followed by surgery (Koshariya *et al*, 2007). Adjuvant chemotherapy for CRC has been desirable in highly suspicious metastatic cases. In these cases, the assessment of *TRIB3* expression may be useful to predict patient prognosis.

In biological assessment, this study showed that *TRIB3* expression was related to tumour growth in several gastrointestinal cancer cell lines. The *in vivo* study showed that siRNA inhibition of *TRIB3* resulted in a reduction in cell growth of seven gastrointestinal cancer cell lines, significantly (*P*<0.05). Although previous reports showed that *TRIB3* is expressed in several cancer cell lines, this study shows that *TRIB3* seems to stimulate proliferation, and may be a new target for the therapy of gastrointestinal cancer (Bowers *et al*, 2003; Xu *et al*, 2007).

Tribs, belong to the pseudokinase family consisting of three mammalian isoforms, Trib1, Trib2 and Trib3, have no detectable kinase catalytic activity because of variations in key amino acids in the ATP-binding domain, but possess substrate-binding domains relating to their function as protein-interacting modules (Seher and Leptin, 2000; Yamatodani *et al*, 2009). Tribs associate with large proteins such as transcriptional factors, and regulate cell growth, differentiation and metabolism (Boudeau *et al*, 2006).

Trib1 interacts with Mapk and modulates Mapkk activity associated with smooth muscle cell proliferation and migration (Kiss-Toth *et al*, 2004; Sung *et al*, 2007). Trib2 has a role in adipogenesis in combination with the degradation of C/EBPbeta (Naiki *et al*, 2007). Trib3 promotes ubiquitination and degradation of proteins involved in cell-cycle regulation and oogenesis through an interaction with activation transcription factor 4, and is involved in the Pten pathway through interaction with Akt (Mata *et al*, 2000; Du *et al*, 2003; He *et al*, 2006; Koh *et al*, 2006; Kato and Du, 2007; Yao and Nyomba, 2008). Trib3 expression is increased in several primary tumours and cancer cell lines and can be controlled by nutrient starvation, which is consistent with these data (Bowers *et al*, 2003; Schwarzer *et al*, 2006; Xu *et al*, 2007). Our report indicates that *TRIB3* is not only a new independent prognostic factor and predictor of metachronous metastasis, but is also a useful target because the inhibition of *TRIB3* may lead to the reduction of CRC through the control of cell growth.

## Figures and Tables

**Table 1:** Clinicopahological factors and *TRIB3* mRNA expression in 202 colorectal cancers

	**High expression**	**Low expression**	
**Factors**	**(%)**	**(%)**	***P*-value**
*Age (years)*			
⩽67	45 (44.5)	51 (50.5)	0.397
67<	56 (55.5)	50 (49.5)	
			
*Gender*			
Male	63 (62.4)	52 (51.5)	0.118
Female	38 (37.6)	49 (48.5)	
			
*Histological grade*			
Wel-Mod	95 (94.1)	89 (88.1)	0.138
Others	6 (5.9)	12 (11.9)	
			
*Tumour size (mm)*			
⩽30	24 (23.8)	22 (21.8)	0.737
30<	77 (76.2)	79 (78.2)	
			
*Tumour invasion*			
Tis	5 (5.0)	10 (9.9)	0.418
T1	6 (5.9)	11 (10.9)	
T2	20 (19.8)	19 (18.8)	
T3	49 (48.5)	44 (43.6)	
T4	21 (20.8)	17 (16.8)	
			
*Lymph node metastasis*			
N0	60 (59.4)	61 (60.4)	0.885
N1–2	41 (40.6)	40 (36.6)	
			
*Lymphatic invasion*			
Absent	49 (48.5)	51 (50.5)	0.778
Present	52 (51.5)	50 (49.5)	
			
*Venous invasion*			
Absent	78 (77.2)	74 (73.3)	0.514
Present	23 (22.8)	27 (26.7)	
			
*Metastasis*			
M0	68 (67.3)	90 (89.1)	<0.001
M1	33 (32.7)	12 (10.9)	

Wel=well differentiated adenocarcinoma; Mod=moderately differentiated adenocarcinoma; Others=poorly differentiated adenocaricinoma and mucinous carcinoma; TRIB3 tribbles homologue 3.

The statistic significance is shown with under line.

**Table 2:** Univariate and multivariate analysis for overall survival (Cox proportional hazards regression model)

	**Univariate analysis**			**Multivariate analysis**		
**Factors**	**RR**	**95% CI**	***P*-value**	**RR**	**95% CI**	***P*-value**
*Age(years)*						
(⩽67/67<)	1.23	0.85–1.80	0.258			
						
*Gender*						
(Male / female)	1.93	0.90–4.47	0.090			
						
*Histological grade*						
(Wel-Mod / others)	1.54	0.36–4.35	0.511			
						
*Tumour size*						
(30</⩽30)	3.70	1.69–15.66	0.001	2.04	0.88–8.79	0.103
						
*Tumour invasion*						
(T3–4 / Tis-2)	11.00	3.28–68.37	<0.001	2.47	0.60–16.82	0.223
						
*Lymph node metastasis*						
(N1–2 / N0)	4.28	2.02–9.63	0.001	1.49	0.65–3.74	0.348
						
*Lymphatic invasion*						
(Present / absent)	2.44	1.14–5.44	0.021	1.43	0.62–3.49	0.396
						
*Venous invasion*						
(Present / absent)	2.17	0.92–4.73	0.071			
						
*Metastasis*						
(M1 / M0)	21.89	9.33–60.11	<0.001	9.34	3.70–27.28	<0.001
						
*TRIB3 mRNA expression*						
(median< / <median)	8.45	2.97–35.48	<0.001	3.78	1.27–16.35	0.014

RR=relative risk; CI=confidence interval; Wel=well differentiated adenocarcinoma; Mod=moderately differentiated adenocarcinoma; Others=poorly differentiated adenocaricinoma and mucinous carcinoma; TRIB3 tribbles homologue 3.

The statistic significance is shown with under lines.

**Table 3:** Univariate and multivariate analysis for metachronous metastasis-free over 5 years survival rate (Cox proportional hazards regression model)

	**Univariate analysis**			**Multivariate analysis**		
**Factors**	**RR**	**95% CI**	***P*-value**	**RR**	**95% CI**	***P*-value**
*Age(years)*						
(67</⩽67)	1.33	0.85–2.09	0.202			
						
*Gender*						
(Male / female)	2.44	0.97–6.90	0.055			
						
*Histological grade*						
(Wel-Mod / others)	24.0	4.78–101.61	<0.001	25.9	3.57–215.84	0.001
						
*Tumor size*						
(30< / ⩽30)	3.66	1.66–15.55	<0.001	3.04	1.18–13.62	0.017
						
*Tumor invasion*						
(T3–4 / Tis-2)	4.80	1.61–20.58	0.003	2.77	0.67–15.00	0.160
						
*Lymph node metastasis*						
(N1–2 / N0)	4.01	1.65–10.26	0.002	2.65	0.98–7.59	0.054
						
*Lymphatic invasion*						
(Present / absent)	4.49	1.73–13.83	0.001	0.72	0.20–2.95	0.637
						
*Venous invasion*						
(Present / absent)	3.10	1.21–7.53	0.019	1.68	0.61–4.53	0.301
						
*TRIB3 mRNA expression*						
(median< / ⩽median)	4.33	1.45–18.59	0.006	3.86	1.09–19.00	0.035

RR=relative risk; CI=confidence interval; Wel=well differentiated adenocarcinoma; Mod=moderately differentiated adenocarcinoma; Others=poorly differentiated adenocaricinoma and mucinous carcinoma; TRIB3 tribbles homologue 3.

The statistic significance is shown with under lines.
