# Supplementary figures and images for: Abnormal expression of TRIB3 in colorectal cancer: a novel marker for prognosis
Source: Br J Cancer. 2009 Nov 10;101(10):1664–70. doi: 10.1038/sj.bjc.6605361 (PMC2778541; doi:10.1038/sj.bjc.6605361)

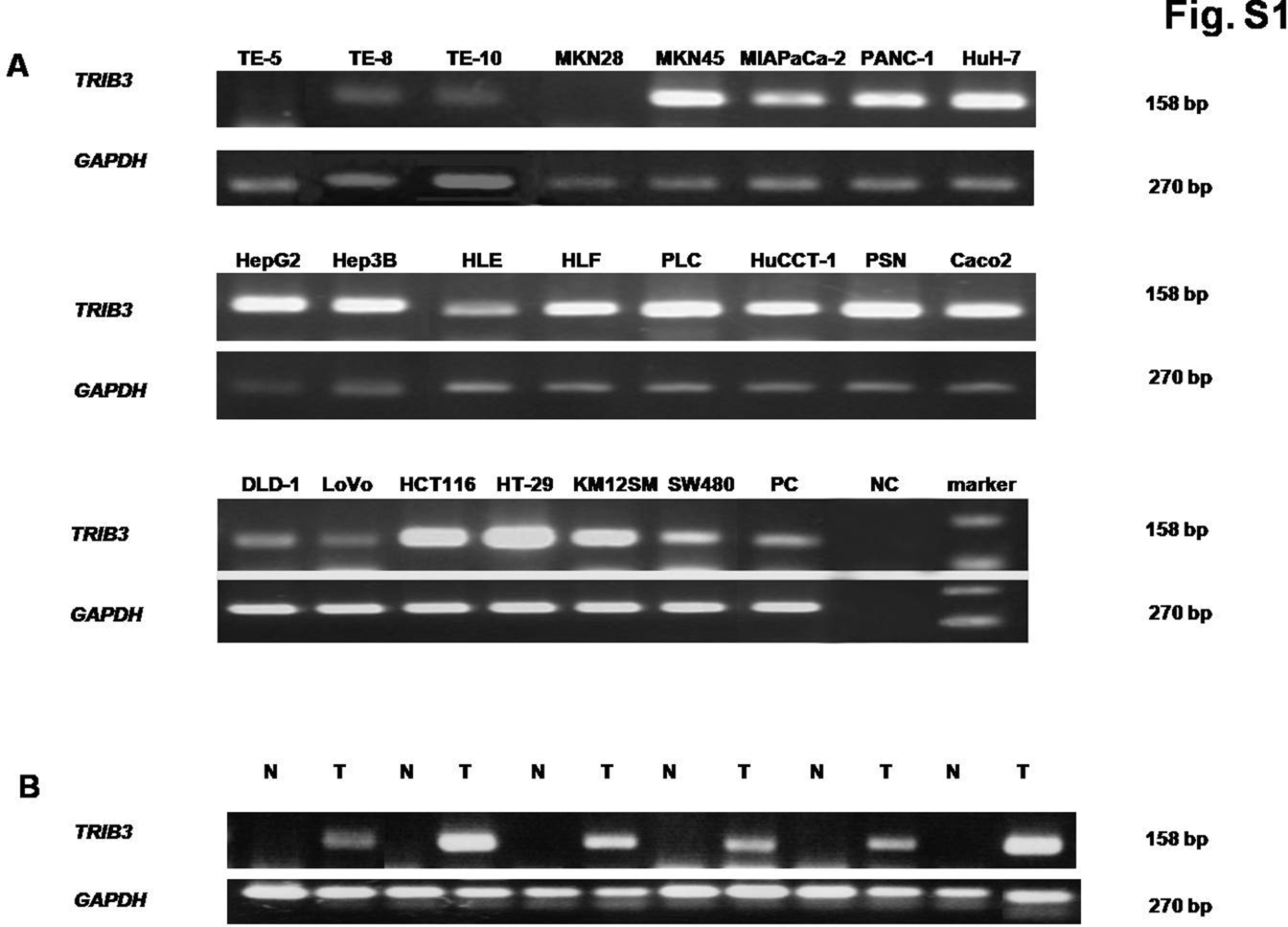

Supplement: Supplementary Figure S1 [file 6605361x1.tif]

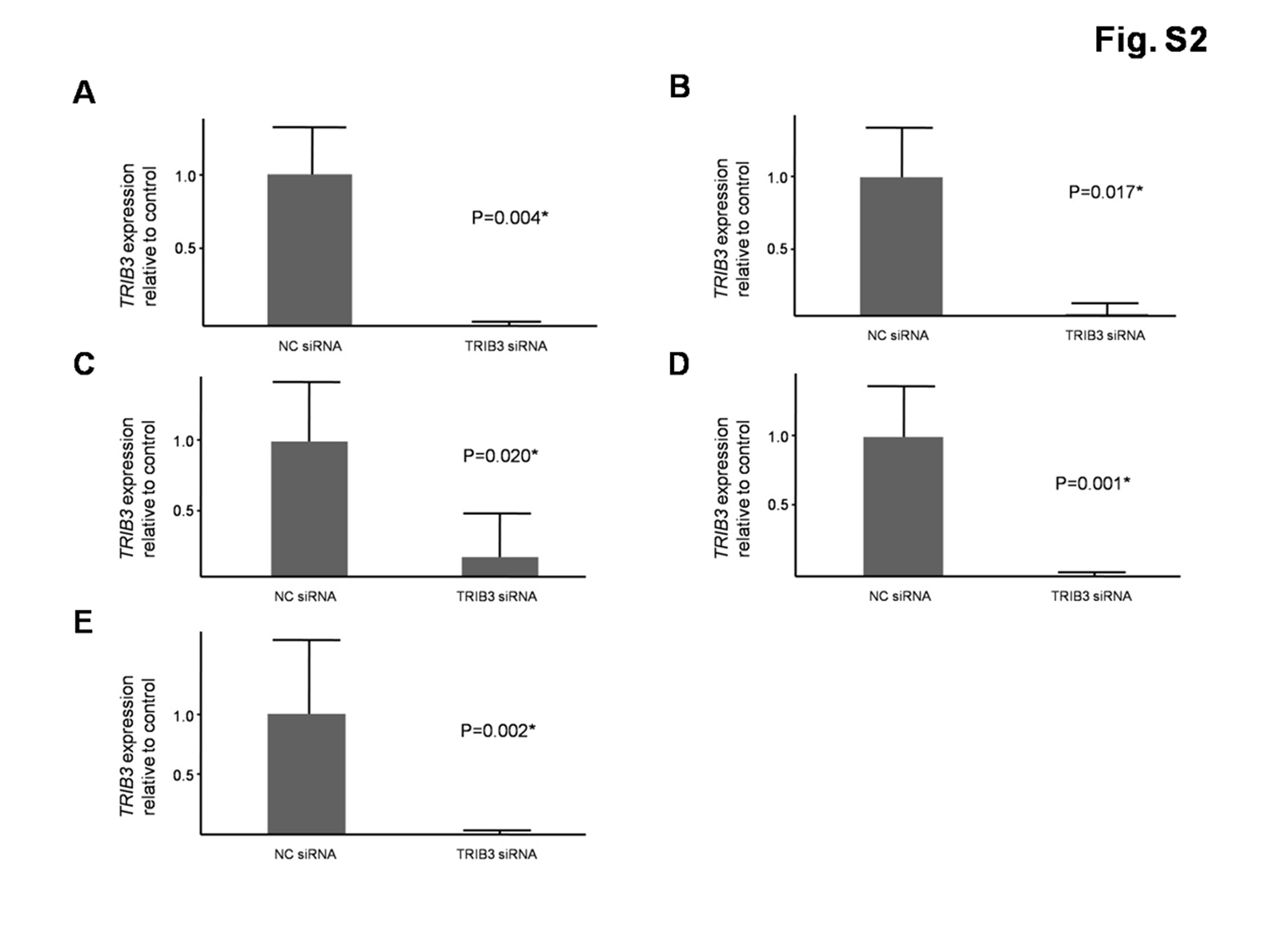

Supplement: Supplementary Figure S2 [file 6605361x2.tif]
