# Supplementary material for: Abnormal expression of TRIB3 in colorectal cancer: a novel marker for prognosis
Source: Br J Cancer. 2009 Nov 10;101(10):1664–70. doi: 10.1038/sj.bjc.6605361 (PMC2778541; doi:10.1038/sj.bjc.6605361)
Supplement: Supplementary Figure Legend [file 6605361x3.doc]

**Supplementary figure legends**

**Fig. S1 *TRIB3* mRNA expression in 22 gastrointestinal cancer cell lines and clinical tissue specimens.**

(A) *TRIB3* gene expression status shown in 20 cell lines derived from human gastrointestinal cancer by RT-PCR analysis. Of the 22 cell lines, 20 (90%) expressed the high levels of *TRIB3*. See text.

(B) RT-PCR analysis of *TRIB3* in CRC and paired normal regions obtained from seven patients. In all seven cases, *TRIB3* expression was higher in cancer regions than in paired normal regions. PC, positive control; NC, negative control.

**Fig. S2 The siRNA inhibition of *TRIB3* in CRC cell lines.**

The suppression of *TRIB3* expression was confirmed by RT-PCR. The reduction was significant in *TRIB3* siRNA experiment, compared with NC (p < 0.05, Student’s t-test) in the 5 cell lines (A, DLD-1; B, LoVo; C, HCT-116; D, KM12SM; E, SW480). NC, negative control.
