# Supplementary material for: Abnormal expression of TRIB3 in colorectal cancer: a novel marker for prognosis
Source: Br J Cancer. 2009 Nov 10;101(10):1664–70. doi: 10.1038/sj.bjc.6605361 (PMC2778541; doi:10.1038/sj.bjc.6605361)
Supplement: Supplementary Information [file 6605361x4.doc]

**Supplementary information**

**Gene Characterization**

To characterize the prevalent CRC in Japanese population, we studied whole genome-wide expression and genome alterations. Data of tissue specimen were collected by laser-captured micro-dissection, as we described previously (*Ann Surg Oncol* 2007; 14:885-92). A total of 88 quality–controlled micro-dissected case of CRC were analyzed using a microarray containing 88 human oligonucleotide probes by the Affymetrix transcriptional profiling (Santa Clara, CA). The global gene expression in primary CRC and corresponding normal regions, and its potential relevance to clinico-pathological characteristics and patient survivals, were estimated.

Genomic array-based comparative genomic hybridization (CGH) experiments were done using the Agilent 44K human genome CGH microarray chip (Agilent Technologies, Palo Alto, CA). Theoretical values of gain or loss of any given region in the genome are denoted as follows: 4 copies, log_2_(2); 3 copies, log_2_(1.5); 2 copies, log_2_(1); 1 copy, log_2_(0.5); and zero copies, log_2_(0). As a result from unexpected noises such as variations on array platform, such as cell variations, heterogeneity of cancer tissues due to asymmetrical mitosis, and contamination of cancer and normal cells during the process of micro-dissection, the estimated values incidentally could be deviated from theoretical values. By confirming unexpected noises are excluded, a total of 63 quality-controlled, micro-dissected cases of CRC were analyzed by CGH microarray, based on the following criteria: high-level amplification (>1.000 at log_2_), gains (0.500 <log2 ≤ 1.000), within normal limits (−0.500 ≤ log_2_ ≤ 0.500), losses of heterozygosity (−1.000 ≤ log_2_ < −0.500) and homozygous deletions (log_2_ < −1.000).

As the study of the etiology, the questionnaire to every patient in this study was carried out, and the correlation between lifestyle and risk-factor of the oncogenesis of CRC were investigated. Results showed no association with *TRIB3* was detected (data not shown). The assessment of transcriptional profiling and CGH data indicated *TRIB3* gene as gain at chromosome 20, which was studied here; the region of *TRIB3* gene showed gains on the CGH in 80 / 88 (90.9%) cases with amplification.

**Clinical tissue samples**

Tumor tissues were extracted immediately after surgical resection. We first recognized tumor margins under operation. Non-cancerous tissues were extracted in the surgical specimen at least 2-cm far from each tumor. Secondly, no contamination of tumor cells in apparently normal tissues was confirmed under microscopic examination, by hematoxilin-eosin, Elastica van Gieso staining and immunohistochemistry, if necessary (anti-PCNA staining; proliferating cell nuclear antigen, a [protein](http://en.wikipedia.org/wiki/Protein) that expresses abundantly in proliferating cells, by a biochemical action [DNA polymerase delta](http://en.wikipedia.org/wiki/DNA_polymerase#Eukaryotic_DNA_polymerases) in [eukaryotic](http://en.wikipedia.org/wiki/Eukaryotic) [cells](http://en.wikipedia.org/wiki/Cell_(biology)); *J Clin Pathol* 1992; 45: 416–419).
